# Modelling human navigation and decision dynamics in a first-person herding task

**DOI:** 10.1098/rsos.231919

**Published:** 2024-10-30

**Authors:** Ayman bin Kamruddin, Hannah Sandison, Gaurav Patil, Mirco Musolesi, Mario di Bernardo, Michael J. Richardson

**Affiliations:** ^1^ Modeling and Engineering Risk and Complexity, Scuola Superiore Meridionale, Naples, Italy; ^2^ Department of Electrical Engineering and ICT, University of Naples Federico II, Naples, Italy; ^3^ School of Psychological Sciences and Performance and Expertise Research Center, Faculty of Medicine, Health and Human Sciences, Macquarie University, Sydney, Australia; ^4^ Department of Computer Science, University College London, London, UK; ^5^ Department of Computer Science and Engineering, University of Bologna, Bologna, Italy

**Keywords:** navigation, decision-making, artificial agents

## Abstract

This study investigated whether dynamical perceptual-motor primitives (DPMPs) could also be used to capture human navigation in a first-person herding task. To achieve this aim, human participants played a first-person herding game, in which they were required to corral virtual cows, called targets, into a specified containment zone. In addition to recording and modelling participants’ movement trajectories during gameplay, participants’ target-selection decisions (i.e. the order in which participants corralled targets) were recorded and modelled. The results revealed that a simple DPMP navigation model could effectively reproduce the movement trajectories of participants and that almost 80% of the participants’ target-selection decisions could be captured by a simple heuristic policy. Importantly, when this policy was coupled to the DPMP navigation model, the resulting system could successfully simulate and predict the behavioural dynamics (movement trajectories and target-selection decisions) of participants in novel multi-target contexts. Implications of the findings for understanding complex human perceptual-motor behaviour and the development of artificial agents for robust human–machine interaction are discussed.

## 1. Introduction

Many everyday activities require that individuals navigate complex and cluttered environments. Whether walking on a busy street, playing team sports or moving around an office space, such behaviour involves navigating towards one or more fixed or moving goal location(s) while simultaneously avoiding stationary and moving obstacles. Traditional approaches to understanding navigational behaviours have assumed that these behaviours are solely dependent on neurocognitive path planning or integration processes and require the construction of representational maps of the task environment [1–3]. However, a growing body of research (e.g. [4–11]) has demonstrated that human navigational trajectories often emerge naturally from the real-time attractive and repulsive influences of goal or subgoal locations and perceived environmental obstacles on an agent’s current heading direction. This research has also shown how the behavioural dynamics [12] of human locomotive navigation and route selection can be effectively modelled using a small set of environmentally coupled dynamical functions [9,12–14], here referred to as dynamical perceptual-motor primitives (DPMPs) [15–18], that capture how information about the relative location of navigation goals and environmental obstacles prospectively modulates an agent’s heading direction during self-directed motion.

In addition to capturing the behavioural dynamics of human navigation, DPMPs have been used to model the perceptual-motor behaviour of individuals across a wide range of task contexts, from simple object reach, collision avoidance and postural control tasks [13,19], to more complex cooperative and competitive interpersonal and team coordination tasks [20–23]. Perhaps one of the best examples of how complex human behaviour can be captured using DPMPs is the research demonstrating how a simple DPMP system can model the behavioural dynamics of human participants completing simulated multi-agent herding tasks [15,24]. For these tasks, pairs of participants are required to control virtual herder agents (HAs) and corral and contain a small herd (4–7) of target agents (TAs, often represented as virtual sheep or cattle) in a specified containment area. The task (or game) is typically presented on a large tabletop display screen, with participants using either a handheld motion tracker or a touch-screen interface to control the movements of the HAs. Repeated studies have shown that with enough practice, all pairs converge on the same modes of perceptual-motor behaviour. Of more relevance here, this research has also validated how corralling movements of participant-controlled HAs can be effectively modelled using a simple, low-dimensional DPMP model [24–27] that attractively couples the location of a participant’s HA to the location of the TA that is (i) furthest from the containment area and (ii) closer to the current location of the participant’s HA relative to the location of the other participant’s HA (e.g. [15,24,28]). Importantly, not only is this elementary DPMP model able to effectively reproduce the behavioural dynamics of human participants, but when integrated into the control architecture of artificial HAs (i.e. model-controlled HAs), results in HA movement trajectories that are not only indistinguishable from human-controlled HA movements [15,24] but also provide the same level of skill training to novice participants [16].

Although the aforementioned herding research has provided compelling evidence that complex human perceptual-motor behaviours can be modelled using simple, low-dimensional DPMPs, the tabletop format of the herding task has raised questions about whether the resultant DPMP models can be generalized to more realistic or real-world navigational task contexts [28–30]; that is, where agents perceive the environment from an egocentric or first-person point of view. Addressing these issues requires investigating and modelling the movement patterns of participants completing the task from a first-person perspective. Consequently, the first objective of the current study was to develop a DPMP model that is capable of capturing the behavioural dynamics of human participants herding TAs using a more realistic first-person herding task.

It is important to appreciate that switching from a tabletop to a first-person herding task is not an incremental extension of previous work, but radically changes the nature of the behavioural control problem. That is, from a hand (end-effector) control problem, where participants have global information about the state of the task environment, to a first-person navigational control problem, where participants only have local information about the task environment limited by their field of vision, different behaviours are expected to emerge. Thus, rather than examining whether the tabletop herding DPMP model could be adapted to a first-person context, we investigated whether the DPMP navigation model proposed by Warren and Fajen and colleagues [4–7,9,12] could be adapted to effectively capture and simulate the navigational movements of a human HA when moving towards and corralling TAs from a first-person perspective. Previous research has shown that a task dynamical approach proposed by Warren and Fajen can be applied to various navigational behaviours such as crowd behaviours [10] and moving targets [7]. However, no previous research has attempted to explore whether the DPMP model of human navigation is able to capture human navigational behaviour in a context where the aim is to guide another autonomous agent (i.e. a TA) to a specified goal location within a task environment.

Another important distinction between the current study and previous research is that when the TA herd size is >1, HAs are required to switch between different navigational (sub)goal locations as a function of which TA is selected to be corralled at any point in time. Therefore, determining whether a DPMP model can effectively model human navigation behaviour in a first-person herding task context also requires identifying which TA-selection policy or decision-making process human participants use when completing the task. Previous research has demonstrated how the effectiveness with which DPMP models are able to capture complex human perceptual-motor behaviour fundamentally depends on the action decision policy used to define what environmental object, surface, event or information the model functions are coupled to at any point in time [17,18,25]. For human behaviour, these action decision policies are typically defined using heuristics or rules-based approaches [15,24]—although see [17,30] and the discussion for how these action decision policies can also be defined using machine-learning techniques. Consequently, the second aim of the current study was to identify which heuristic TA-selection policies human participants employed to complete a first-person herding task. Of particular interest was whether human herders would converge on the same policy (or set of policies) and what informational variables defined the policies employed.

**Figure 1.**
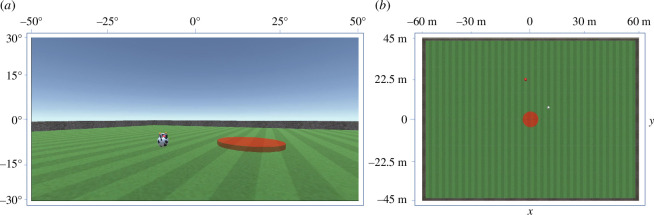
Task environment. (*a*) Example of a participant/HA’s first-person view of the game field. The TA (spherical cow with head) and sections of the containment zone (red) and the exterior walls of the field (grey) are also visible. (*b*) A bird’s-eye view of the game field (not seen by the participant), where the HA is the small red dot and the TA is the small white dot.

In summary, the current work has two aims. The first aim was to model the navigational movement dynamics of HAs engaged with single TAs in the herding task. The second aim was to identify the target-selection (TS) policy (or policies) that human actors employed when completing a first-person herding task. To achieve these aims, the tabletop herding task previously used by Nalepka *et al*. [15,24] was translated into a first-person herding video game, in which human participants controlled a virtual HA from a first-person point of view using a keyboard and mouse to find and corral one or more TAs into a containment zone located in the centre of a large three-dimensional (3D) game field (see figure 1). Two studies using this first-person herding game were then conducted to address the two research aims (i.e. research questions). The first study—single-target herding—was designed to address the first aim. More specifically, it explored how participants corralled a single TA into the containment zone, in order to first develop a DPMP model capable of replicating the movement trajectories exhibited by participants when approaching and corralling a TA into the contained zone. The second study—multi-target herding—addressed the second aim by exploring how participants selected and corralled multiple targets into the containment zone. Two experiments were conducted to first identify the TA-selection policy(s) employed by participants and then use the predominating TA policy to validate whether the DPMP model developed in the single-target herding experiment could be generalized to a multi-target context.

Given the highly interconnected nature of the two studies and the research questions they address, we present our findings in turn. For the single-target study, we first detail the results and analysis of the movement trajectories exhibited by human participants, followed by details about the model developed to capture these movement dynamics, and the results of model validation and simulations. We then turn to the results for the multi-target herding study. This includes an analysis of the different potential and most often adopted TS policies human participants employed, as well as validation tests of the final multi-target herding model. This is followed by a combined discussion of the results of both experiments, with the detailed methods employed for both studies provided in the final section of the paper.

## 2. Single-target herding

As stated above, the aim of the first study was to identify and model the movement trajectories of human participants as they approached and then corralled a TA into the specified task containment zone (see figure 1). More specifically the aim was to examine whether the navigational model proposed by Fajen & Warren [4,5] could be adapted to capture the behavioural dynamics of human herders (participants) completing a first-person herding task.

To achieve this aim, the movement and target approach trajectories of 24 participants, who were required to corral a single target into the containment zone, were recorded and analysed. Participants completed 20 trials in total (5 practice and 15 experimental). They were instructed to complete the trials as quickly as possible, receiving no additional guidance beyond information on how to control their HA using the keyboard and mouse. At the beginning of each game trial, the (participant-controlled) HA and the TA started off at rest at locations pre-determined by a specified set of initial conditions, with these conditions randomized in order across participants. Importantly, the TA was repelled (moved directly) away from the participant-controlled HA when the HA came within 10 metres of the TA. The TA would gradually come to a stop if no longer influenced by an HA. Each game trial ended when the participant effectively ‘chased’ the TA into the containment area, that is, when the TA would come to a halt inside the containment zone (see §6 for a detailed description of HA control, TA movement dynamics and experimental procedures employed).

### 2.1. Results

#### 2.1.1. Participant movement trajectories

**Figure 2.**
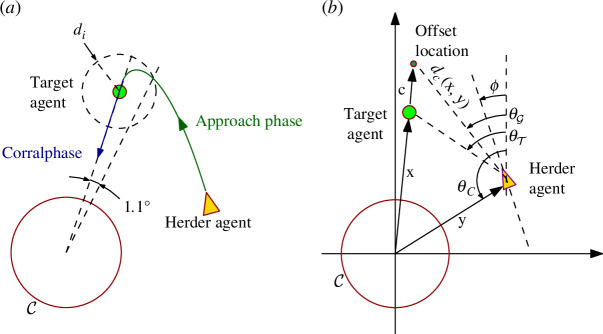
(*a*) The phases of the herding task. The HA first approached the TA and then switched to corralling it towards the containment zone C. The median angle at which human HAs first influenced TA was 1.1°. $d_{i}$ is the influence radius of the TA, equal to 10 m. (*b*) The model variables: C denotes the containment zone, 𝐱 the position of the TA, 𝐲 that of the HA. 𝐜 is the offset from the TA, $d_{c}(𝐱,𝐲)$ the distance between the offset goal location and the HA. ϕ is the HA heading angle and $\theta_{G,T,C}$ the angles of the offset goal location, TA and C, respectively (measured from the vertical axis). See the text for more details.

An analysis of participant data revealed that (human) HA trajectories consisted of the following two distinct phases: (i) an approach phase and (ii) a corral phase (see figure 2*a*). During the approach phase, participants moved the HA to a position behind the TA with respect to the containment zone. That is, all participants navigated towards an offset location that was a distance c away and behind the TA with respect to the containment zone. For this first phase of behaviour, participants also typically maintained a distance greater than the HA–TA repulsion distance to ensure that the TA was not pushed further away from the containment zone.

For the second, corral phase, participants moved directly towards the TA entering the TA influence region in order to repel (drive) the TA in a (more or less) straight line towards the containment zone. Importantly, the angle at which the HA began influencing the TA could be used to differentiate the two phases. Furthermore, the median of these angles across all participants, measured as the angle between the positional vectors of the HA and the TA originating from the centre of the containment zone, corresponded to 1.1° (figure 2*a*), indicating that the participants transitioned to corralling just before being perfectly aligned (on a straight line) with the TA and the centre of the containment zone. Finally, the participants never (except for one out of 360 trajectories) passed through the containment zone (even though they could and were never given instructions either way).

#### 2.1.2. Herding navigational model

To model the navigational trajectories of the participants during the task, the approach and corral phases were modelled using a modified version of the navigation model proposed in a study by Fajen & Warren [4]. Specifically, assuming constant forward motion with $v(t)=v_{0}$ the HA speed, i.e.


(2.1) $$\begin{array}{ccc} & \|\dot{𝐲}\|=v(t)=v(0)=v_{0}, & \end{array}$$

and an exo-centric reference frame, the heading direction ϕ of an HA was modelled (see figure 2*b* for set-up of variables) as


(2.2) $$\begin{array}{rl} \ddot{\phi } & =-b\dot{\phi }+\psi_{G}(x,y,\phi ,\theta_{G})+\psi_{T}(x,y,\phi ,\theta_{T})\\ & +\psi_{C}(x_{C},y,\phi ,\theta_{C}), \end{array}$$

where $b\in {\mathbb{R}}^{+}$ is a positive damping constant and $\psi_{G}(𝐱,𝐲,\phi ,\theta_{G})$ represents the attractive coupling between the forward heading direction of the HA and the angular position of the target-offset location. Again, this offset target location corresponds to the location behind the TA and is the goal location that the HA is attracted to. Moving towards this offset location, rather than the location of the TA itself, ensured that the HA did not influence the TA during the approach phase.

To further ensure that the HA does not influence the TA during the approach phase, the term $\psi_{T}(𝐱,𝐲,\phi ,\theta_{T})$ in equation (2.2) provides a repulsive coupling between the HA heading direction and the TA’s angular position with respect to that heading. This repulsive force disappears smoothly as the HA moves around and behind the TA (with respect to the containment zone) to allow the HA to approach and drive the TA into the containment zone.

Finally, the term $\psi_{C}({𝐱}_{C},𝐲,\phi ,\theta_{C})$ in equation (2.2) creates a repulsive coupling between the direction of the HA and the centre of the containment zone C, fixed at ${𝐱}_{C}=𝟎$. This was implemented to ensure that the HA avoids getting too close or entering that containment zone, similar to what was observed for the human participants. The full mathematical definitions of each of these terms are presented in §6.

#### 2.1.3. Model validation

In order to determine whether equation (2.2) could effectively capture observed human data (i.e. produce simulated HA movement trajectories equivalent to participant-controlled HA movement trajectories), the model was first parametrized using 12 of the 15 experimental trials and then validated (tested) against the remaining three trials. As detailed in §6, model parametrization (optimization) was conducted by minimizing the normalized (dynamic time warped (DTW) [31,32]) distance between simulated trajectories and the mean human trajectory for each of the 12 parametrization trials (i.e. the corresponding 12 different initial conditions). From the trial-optimized parameters, the median parameter settings were then calculated and used for model validation.

**Figure 3.**
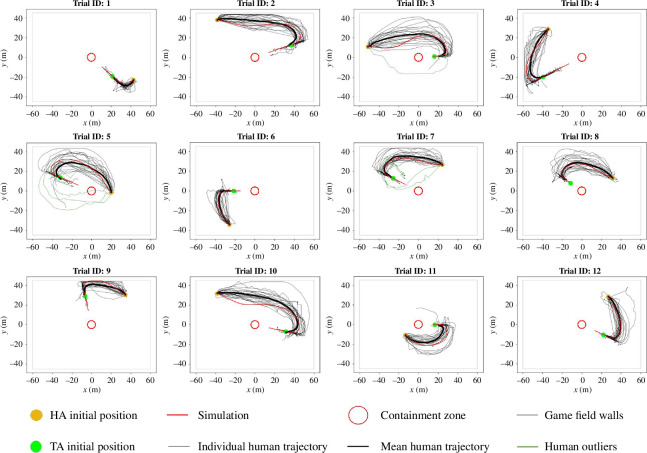
Model training set. The six dropped trajectory outliers excluded from the model training are included in the figure (see §6.3.2 for more details).

**Figure 4.**
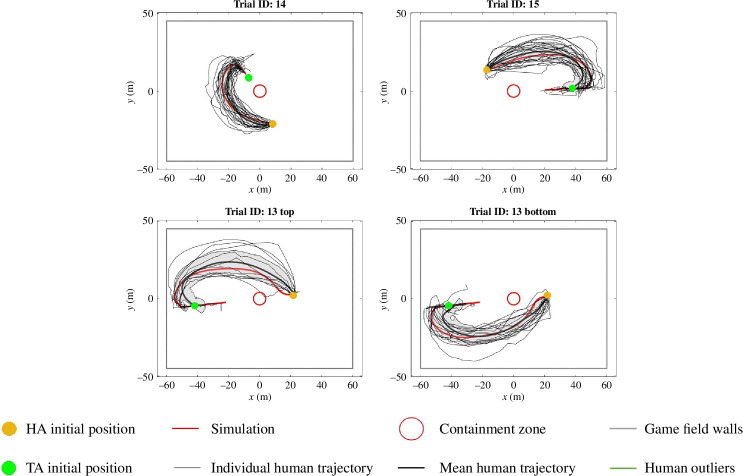
Model test set. Trial ID 13 was the one split into two subtrials, as participants were divided according to whether they took the top or bottom route. The shaded area in grey is the s.d. bound around the mean human trajectory.

As can be seen in figures 3 and 4, these median parameter values produced simulated trajectories that were equivalent to the average human trajectory for each of the 12 parametrization trials and the three test trials, respectively. The trajectories were also representative of the general distribution of the trajectories observed by the participants for each trial. To further validate the similarity of the simulated and mean participant trajectories for the three test trials, four different measures of trajectory similarity were employed (see table 1). The first two were *navigation time*, calculated as the time of the approach and corral phase, and *path length*, calculated as the length of navigation during the approach and corral phase, without observed differences between the simulated and mean human trajectories (both t(6)>0.25,p>0.15), with null effect Bayesian factors of $BF_{10}=0.534$ and $BF_{10}=1.045$ for the path length and navigation times, respectively.

**Table 1.** Trajectory characteristics of human participants and simulations for the four sets of initial conditions unseen by the model during parametrization. Comp. human denotes comparative human, which is the 90th-percentile furthest human trajectory from the mean human trajectory as per the DTW metric. Sim denotes simulation. The mean and s.d. columns for the first two characteristics are for human data. The last row provides the mean for all trials.

	path length (m)			navigation time (s)			coverage percentage (%)		error from mean (m)	
trial ID	mean	s.d.	sim	mean	s.d.	sim	comp. human	sim	comp. human	sim
14	72.1	16	70.2	17.8	6.2	18.8	29.8	97	5.829	1.866
15	101.1	17.7	108.9	24.8	4.8	37.7	23.2	83.3	6.184	5.255
13 top	119.2	22.7	126.6	28.4	4.9	42.7	26.4	69.9	8.257	7.138
13 bottom	121.4	20	126.1	29.5	5.3	42.5	47.3	70.7	5.571	5.112
averages	103.5	19.1	108	25.1	5.3	35.4	31.7	80.2	6.46	4.843

The third measure, *coverage percentage*, captured the percentage of a given trajectory that was within the 90% confidence interval (see §6 for more details) around the mean human trajectory. Consistent with the simulated trajectory being equivalent to a normative human trajectory, the simulated trajectories had a consistently higher coverage percentage than the 90th-percentile human trajectory for all initial conditions in the test set (the 90th-percentile human trajectory corresponds to the 90th-percentile furthest human trajectory from the mean human trajectory as per the DTW distance). The fourth measure was the distance from DTW (or *error*) of the simulated trajectories from the mean human trajectories. Again, this error was less for the simulated trajectories compared to the 90th-percentile human trajectory for each test trial, further indicating how the model was able to generate navigational trajectories similar to and largely indistinguishable from those exhibited by the participants.

## 3. Multi-target herding

Although the results of the single-target study validating that equation (2.2) could successfully simulate the behavioural dynamics—including the approach and corral phases—of human-controlled HAs when herding a target, of additional interest was the extent to which the model could, once paired with an appropriate TS policy, accurately capture the behaviour of human participants required to corral multiple TAs (i.e. small TA herds). Addressing this research aim required identifying the TS policy (or policies) that human participants use to successfully corral a small herd of TAs into the containment zone. As will be explained in more detail below, the results revealed that the majority of human TS decisions could be captured using a single heuristic (rule-based) decision policy. Two experiments were conducted to uncover this. The first involved an analysis of the TS behaviour of the participants when tasked with corralling a herd of three TAs into the containment zone. This was done by analysing the TA-selection sequence of participants across 24 trials (6 practice and 18 experimental). The second experiment involved validating the inferred TS policy by observing the behaviour of a different set of participants across a set of 24 trials (with the initial six practices) where the initial conditions (i.e. the relative location of TAs in a herd) were specifically designed to test the prediction of the inferred policy against possible alternatives.

In this multi-target herding experiment, the game set-up mirrored that of the single-target experiment, except that there were three targets instead of one. Participants were also required to complete five single-target trials before beginning the multi-target trials to familiarize themselves with the game mechanics. These initial single-target trials were also considered practice and were excluded from further analysis (see §6 for more details).

### 3.1. Results

#### 3.1.1. Highest-ranked policies

A preliminary analysis of the data revealed that the TA-selection decisions of the participants reflected three heuristic rules. First, participants preferred to select the TAs that were closest to them in angular rather than linear distance. Here, the angular distance between a given TA and HA was measured with respect to the centre of the containment zone (which was at the origin), and the linear distance was the Euclidean distance on the plane. Second, participants selected successive TAs based on which TA was closest in angular distance to the previous TA selected. This approach was contrasted with the selection of TAs on the basis of their angular distance from the HA’s initial (starting) position. Finally, if two TAs were collinear or almost collinear with respect to the containment zone (within a certain angular distance from each other, approx. 18.9°, with respect to the centre of the containment zone), the participants preferred to select the TA that was further away from the centre of the containment zone, even if that TA was further away from the HA in terms of angular distance.

This three-rule policy, termed the *successive collinear angle* or SCA, was able to predict the TA-selection order exhibited by the participants in 78.8% of the trials. Interestingly, the collinear aspect of this policy meant that participants minimized game effort since when two (or potentially three) TAs were close to collinear with the containment zone, it was more efficient (given the constraints of the task) for participants to influence the TAs that were furthest from the containment zone first, and then influence the second furthest TA en route. That is, participants could corral both near-collinear TAs simultaneously instead of corralling one all the way to the containment zone before repeating this step for the following TA(s).

To validate that SCA captured the majority of the participant’s TA-selection behaviour, other TS policies defined in §6 were also tested, with their prediction scores reported in table 2. In short, these policies tested a variety of possible rules and combinations of rules with regard to (i) distances from the HA or from the containment zone, (ii) linear versus angular distances from the HA, measured from the initial HA position, as well as successively and dynamically (in real time), and (iii) identification of whether the TAs were nearly collinear with the containment zone or not. A two-phase process was used to determine whether SCA better predicted the sequence in which participants corralled TAs compared to other possible policies. In the first phase, the number of times a policy correctly predicted a participant’s target order sequence was recorded in a non-exclusive manner. This was done for the 21 participants for the 18 initial conditions, totalling 378 trials across the entire sample. The precision of each policy was then calculated as a proportion of this total. Given that the policies were not always mutually exclusive (i.e. several policies would correctly predict the same sequence depending on the specific herder-target environmental conditions), for the second phase the target order sequence a participant exhibited on a given trial was then re-classified in a mutually exclusive, stepwise manner, consistent with the policy order rank. To do this, the target order sequence for a given trial was checked to see if it was consistent with the most accurate policy (determined from the non-mutually exclusive policy analysis). If so, it was assigned to that policy, and no further classification checks were performed. If not, it was checked against the next policy in the rank until a consistent policy was found. A target order sequence was classified as ‘other’ when no policy was consistent with that sequence.

**Table 2.** Total count and proportions of policy evaluations across mutually exclusive and non-mutually exclusive categories. The total number of TA-selection policies for participants is the number of participants × number of trials = 21 × 18 = 378. Excl. *N* is the total number of trials of mutually exclusive policies tested, excl. *P* is the respective proportion. Non-excl. refers to the same but for the non-mutually exclusive.

policy	excl. *N*	excl. *P*	non-excl. *N*	non-excl. *P*
successive collinear angle	298	0.788360	298	0.788360
furthest from containment zone	24	0.063492	71	0.187831
successive closest angle from herder	15	0.039683	242	0.640212
other	10	0.026455	0	0.000000
successive furthest distance from herder	10	0.026455	33	0.087302
dynamic collinear angle	8	0.021164	165	0.436508
furthest distance from herder	6	0.015873	0	0.000000
closest distance from herder	4	0.010582	128	0.338624
closest from containment zone	2	0.005291	30	0.079365
dynamic collinear distance	1	0.002646	156	0.412698
successive closest distance from herder	0	0.000000	182	0.481481
successive furthest angle from herder	0	0.000000	25	0.066138
dynamic furthest distance from herder	0	0.000000	0	0.000000
dynamic closest angle from herder	0	0.000000	162	0.428571
dynamic furthest angle from herder	0	0.000000	0	0.000000
furthest angle from herder	0	0.000000	0	0
closest angle from herder	0	0.000000	0	0
initial collinear angle	0	0.000000	252	0.666667
initial collinear distance	0	0.000000	224	0.592593
successive collinear distance	0	0.000000	278	0.735450
dynamic closest distance from herder	0	0.000000	147	0.388889

This analysis revealed that after SCA, only two other policies, the furthest from the containment zone and successive closest angle from herder, resulted in mutually exclusive accuracy scores greater than 3.0%, specifically 6.34% and 3.96%, respectively. Regarding non-mutually exclusive accuracy, the next-best policy was successive collinear distance, which entails the same three rules as SCA but is defined in terms of linear (planar) distance. Another policy, initial collinear angle, differs from SCA in that the TA order is based on the angular distance of TAs from the HA’s initial position. Finally, the fourth-best policy, the successive closest angle from herder, entails the first two rules of SCA, but not the third collinear rule.

#### 3.1.2. Policy validation

**Figure 5.**
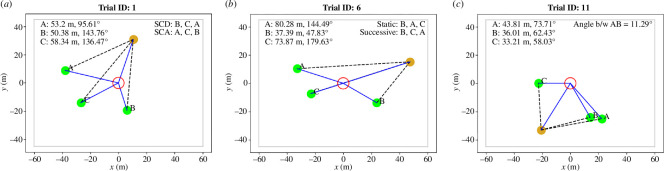
Initial conditions were designed to specifically test leading policies. Each test would confirm or negate the validity of SCA against competing subsuming policies. (*a*) Comparing the successive collinear distance and SCA, (*b*) comparing the initial collinear angle and SCA and (*c*) examining the relationship between successive angle and SCA. TAs are labelled A, B and C, and their distances from the HA, including the angle of the HA subtended at the centre of the containment zone, are indicated. The expected order of the TA engagement according to the competing tested policies is also included for the first two plots. In (*c*), it was found that individuals had different thresholds for cluster identification, so the expected policy outcome is not included here but is detailed in §3.1.2.

Given the similarity in the top four non-mutually exclusive policies, a subsequent policy validation experiment was conducted, in which participants completed a specific set of trials that were designed to better assess whether participants’ TA selections were most aligned with SCA. Figure 5 presents examples from the set of validation initial conditions that were specifically designed to test whether the TA selections were better predicted by (i) linear versus angular distance, (ii) successive versus initial angular distance, and (iii) collinear clustering or its absence. A full presentation of the initial conditions used for the validation is available in the electronic supplementary material.

As expected, SCA was confirmed as the policy that predicted most of the human TA-selection orders, with a prediction accuracy over the entire set of validation trials of 71.7%. With regard to subsets of validation trials, SCA more accurately predicted participant behaviour compared to all other alternative and competing policies tested. In the initial conditions testing the angular versus linear distance, the SCA had a score of 78%, while successive collinear distance predicted 22% of the trials (only non-mutually exclusive scores are reported in this subsection). Where successive TA selection was tested against initial condition TA selection, SCA scored 78%, while initial condition TA selection scored 12%. Finally, in trials that tested collinear cluster identification, SCA (using a collinear cutoff of 18.9°) obtained a score of 35%, while the successive closest angle predicted only 1% of the trials.

It is important to note that the considerably lower SCA score in this case compared to its overall score of more than 70% was due to the variation in the participant threshold for cluster identification. In the validation trials where the TAs clearly did not form a group (the minimum angle between them, subtended in the centre of the containment zone, was greater than 30°), the participants selected the TA closest in angle 84% of the time as their choice of the first TA and then preferred the SCA policy when choosing the second TA to corral 93% of the time. Furthermore, for the tests in which the TAs potentially formed a group (angle between two or more TAs less than 30° from the containment zone), the participants chose the TA farthest from the containment zone within that cluster 71% of the time. These percentages vary slightly after changing this 30° parameter (for example, after setting it to 25°, this last score increases to 74%), indicating that participants have varying thresholds to classify TA as part of a group. To further illustrate this, the accuracy of the SCA was calculated for each participant in the validation dataset by varying the threshold angle for cluster identification. The thresholds for most participants to treat TA as a group ranged between 15° and 25°.

#### 3.1.3. Model generalization and simulations

As a final validation of the SCA policy and equation (2.2), artificial HA simulations were performed, where the control architecture of artificial HA included equation (2.2) and the SCA TS policy. Simulations were carried out using the same trial conditions used for the multi-target herding experiment detailed in §3. Note that equation (2.2) was slightly modified so that non-targeted TAs (the TAs that are not currently chased) were treated as standard obstacles (see §6 for more details).

**Figure 6.**
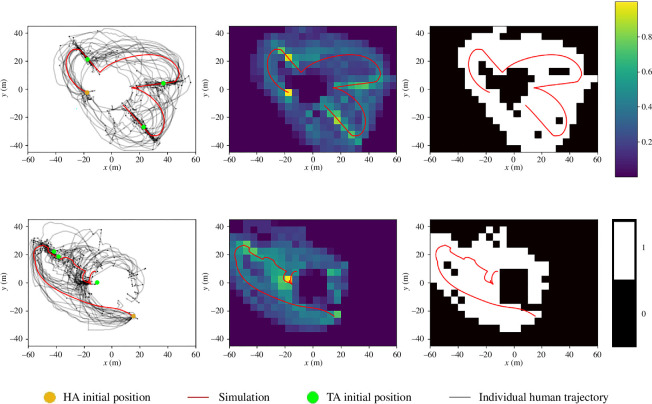
Plots of simulations, human data and evaluation methods. The first panel on the left displays both human and simulated trajectories (legend consistent with other two-dimensional trajectory plots in this article). The upper plot demonstrates the selection of TAs based on the closest angular distance on a successive basis (‘successive angle’) and the lower plot illustrates the selection based on angular proximity, chosen successively (‘SCA’). The second panel overlays the simulation onto a nonlinear heatmap representing the spatial frequencies of human trajectories. The third panel features the simulation overlaid on the binary map derived from the nonlinear heatmap. Colour bars indicating the relative spatial frequencies of the nonlinear heatmap and the binary map are included in the final panel.

As expected, our DPMP model combined with the SCA TS policy generated simulated HA trajectories that were consistent with the observed human data. The similarity between the simulated and participant data was determined using weighted and binary trace maps, examples of which are provided in figure 6. Simply stated, weighted and binary trace maps represent the areas on the movement plane most frequented by participants (see §6 for more details), with the white area in the binary trace maps approximating the confidence interval. As can be seen from an inspection of figure 6 and table 3, the simulated HA behaviour consistently fell within the prototypical trajectory areas of the participants, producing sequences of HA trajectories that represented the mean human trajectory. Figures of all model fits and binary and weighted trace maps are presented in the electronic supplementary material.

**Table 3.** Binary traces.

trial	mean	s.d.	sim	trial	mean	s.d.	sim
1	0.93	0.06	0.98	1	23.55	2.06	25.34
2	0.94	0.05	0.98	2	21.87	1.83	24.34
3	0.90	0.09	0.98	3	23.38	2.96	25.99
4	0.95	0.05	1.00	4	24.48	1.97	27.25
5	0.92	0.07	1.00	5	24.27	1.66	27.59
6	0.93	0.08	0.97	6	23.38	2.68	25.32
7	0.91	0.08	0.96	7	22.42	1.65	24.34
8	0.92	0.10	0.98	8	23.45	2.71	25.24
9	0.93	0.07	1.00	9	24.31	2.04	26.73
10	0.94	0.05	0.92	10	23.85	1.85	25.15
11	0.92	0.08	0.93	11	22.51	2.29	24.40
12	0.94	0.06	0.94	12	25.70	2.24	27.11
13	0.95	0.07	1.00	13	24.53	3.11	28.22
14	0.92	0.07	1.00	14	23.88	2.42	25.48
15	0.93	0.08	1.00	15	23.54	1.95	27.59
16	0.94	0.08	0.87	16	24.66	2.91	23.86
17	0.92	0.06	0.88	17	21.05	1.99	22.11
18	0.94	0.04	0.98	18	23.92	1.62	25.79
average	0.93	0.07	0.97	average	23.6	2.22	25.66
(a) binary traces				(b) weighted traces			

## 4. Discussion

The objective of the current study was to further explore whether DPMPs can be utilized to capture complex human navigation behaviour, specifically within the context of a first-person herding task. Two experiments were carried out in which participants controlled the navigational movements of a virtual HA to corral one or more virtual cows (here referred to as TAs) into a containment zone, located at the centre of a large game field.

In the first experiment, the single-target herding experiment, an analysis of participants’ HA movement trajectories revealed that trajectories consisted of the following two distinct phases: (i) an approach phase, in which participants moved the HA to an offset position behind the TA, with respect to the containment zone; and (ii) a corral phase, where, after reaching the target-offset location, participants moved directly towards the TA, entering its influence region, to guide the TA in a straight line towards the containment zone. More importantly, computer simulations revealed that these behaviours could be captured using an adapted version of the Fajen & Warren [4,6,9] navigation model, in which the HA’s heading direction was simply attracted towards a 6.5 m offset location positioned behind a to-be-corralled TA while treating the to-be-corralled TA as an obstacle. The transition between the approach and corral phases was simply achieved by incorporating a specific function that smoothly reduced the repulsive force between the target and the HA’s heading direction as the HA moved around and behind the TA.

The second experiment, the multi-target herding experiment, involved a more complex herding scenario where participants were required to corral three targets randomly placed around the game field. In addition to examining whether the newly proposed DPMP navigation model could be generalized to a multi-target task context, this experiment aimed to determine the TS policy or policies that participants employed to complete the task. An analysis and subsequent validation experiment of the order in which participants corralled the targets revealed that participants employed the same TS policy in almost 80% of the trials. This policy, termed the SCA policy, involved three heuristic rules. First, participants chose targets closer to them in angular distance measured with respect to the centre of the containment zone (rather than linear Euclidean distance). Second, they choose successive TAs nearest in angular distance to the previously chosen TA. Lastly, if two (or more) TAs were collinear or nearly collinear with respect to the containment zone (that is, perceived to be part of the same corral cluster relative to approaching the containment zone), then participants corralled the TA farther from the containment zone first, even if it was more distant from them in angular terms.

Collectively, the results provide further evidence that the behavioural dynamics of human perceptual-motor behaviours can be effectively modelled using simple, low-dimensional DPMPs [12,14,19,33]. With regard to multi-agent herding, specifically, the current study significantly extends the previous research by being the first study to develop a DPMP navigation model that can be generalized to first-person task contexts. Prior to this study, DPMP models had only been proposed for tabletop herding tasks or where humans (or HAs) had global information about the task context [15,24]. Obviously, more work is required to fully determine the degree to which the current model can be generalized to herding scenarios that entail larger numbers of targets, as well as two or more herders. This is currently being investigated using a multi-player version of the first-person herding game employed here, as well as using an online multi-player video game called ‘Desert Herding’ [34–37] in which teams of three or more players cooperate or compete with other teams to identify and contain large numbers of autonomous robots scattered around a large desert game field. Future research examining whether the proposed model can be generalized to real-world task contexts will also be required, including real-world herding, crowd control, search and rescue and sports-related settings. However, given the recent research by Warren *et al.* demonstrating how similar DPMP navigation models can effectively capture human behaviour in various real-world pedestrian and social settings [38–40], it is likely that the current DPMP model will also generalize to numerous real-world multi-agent herding and corralling task contexts.

The current study also highlights the degree to which DPMP models can effectively capture complex human behaviour, including the enactment of subtask goals, which is fundamentally dependent on identifying and integrating effective action decision policies within the DPMP control architecture [18,29,41]. Indeed, in order to effectively reproduce or predict the behavioural dynamics of participants in the multi-target task scenarios investigated here, the complete DPMP + TS policy model was required. Patil *et al*. [16,17,42] have referred to such models as hybrid-DPMP models and, consistent with a complex systems approach, have argued that it is the reciprocal (co-determinant) interaction of simple movement functions and simple task-relevant action policies that result in hybrid-DPMP models producing remarkably ‘human-like’ patterns of behavioural action—that is, the emergence of task-specific and often complex perceptual-motor behavioural emerges from environmental coupled, non-complex rules.

Regarding the SCA policy identified here, the straightforward nature of the policy and the robustness with which it predicted behaviour across participants, initial conditions and player experience (i.e. over trials) is a testament to the tendency of individuals to converge upon the simplest action policies that result in near-optimal and efficient behavioural performance [30,43–45]. Interestingly, the SCA policy is remarkably similar to the target-selection policy that the majority of participants employ in the tabletop herding tasks [15,28], with the exception that individuals have to continuously select and reselect new targets in the tabletop herding task due to the continuous motion of targets. Moreover, while the current study has assumed that TAs move deterministically in response to approaching HAs (see equation (6.1)), this other tabletop shepherding work [15,28] has considered noisy TA dynamics. Thus, given that similar polices seem to be employed in both situations, the current work further emphasizes the potential generalizability of simple SC-like TA-selection policies. Whether this policy can also generalize to evasive TAs is an additional question and is the subject of current research [34,35]. Given that participants appear to employ similar policies in both first- and global-information settings implies that the SCA policy could reflect a general policy of TS, one that could be employed across a wide range of TS tasks. Recent research by Auletta *et al*. [25,41], which demonstrated how a similar policy can be employed by simulated HAs in contexts with 20+ TAs and three or more herders, provides support for this assertion.

Finally, while we only investigated different heuristic TS policies here, such policies can also be developed using various machine-learning techniques. For instance, using the tabletop herding task paradigm, Rigoli *et al*. [16] have demonstrated how TS policies can be developed using deep reinforcement learning. However, this research also demonstrated that although hybrid DRL–DPMP models result in better simulated task performance, they do not generalize well to human–artificial agent contexts. That is, human participants perform better and prefer to play with heuristic-DPMP models compared to DRL–DPMP models. A method of developing action selection models that better align with human behavioural expectations is using supervised machine-learning (SML) techniques, where action decision policies are developed (trained) using real human action decision data. Auletta *et al*. [30] have recently demonstrated the potential utility of the SML approach, by demonstrating how artificial neural network models can be trained not only to predict the action decisions of participants completing the tabletop herding task but in combination with explainable artificial intelligence (AI) techniques can also be employed to uncover the key information that participants use to make effective action decisions. Motivated by these recent findings, research is currently underway to validate the SCA policy identified here using the SML and explainable AI approach proposed by Auletta *et al*. [30] and to compare the utility of hybrid heuristic-DPMP versus SML–DPMP models for the development of artificial herders for human–machine teaming and training.

## 5. Conclusions

This study marks a significant advancement in our comprehension of human navigation and decision-making when performing a complex task such as herding, showcasing the effectiveness of DPMPs in modelling such complex activities. The research successfully highlights the versatility of DPMPs in handling both single- and multi-target herding tasks. Furthermore, it brings to light the pivotal role of heuristic policies in simulating human decision-making within perceptual-motor tasks. These insights not only broaden the scope of hybrid-DPMP applications to include first-person task contexts—an area previously uncharted—but also indicate the potential for these models to be adapted to real-world scenarios, including crowd dynamics [10,46], evacuation modelling [47,48], virtual reality training systems for firefighters [49], search and rescue and military operations [50–52], as well as environmental cleanup operations such as oil spills [53]. Finally, this study also lays a foundation for future explorations of multi-player or multi-agent first-person herding and other cooperative human perception-action tasks, underscoring the critical need for incorporating effective action decision strategies within DPMP frameworks to accurately replicate the complex behavioural dynamics of human action and interaction.

## 6. Methods

### 6.1. Apparatus and task

The herding task required human participants to control an HA, from a first-person point of view, in order to find and corral either a single (single-target herding experiment) or three (multi-target herding experiment) TAs into a specified containment zone (circular red area) positioned in the centre of a large game field. Example views of the herding task environment are displayed in figure 1, including a screenshot of the first-person point of view of the participant (figure 1*a*), with a single TA (virtual cow) and red containment zone in view, as well as a bird’s-eye view of the entire task environment (figure 1*b*). The game was designed using the Unity game engine (v.3.3.0, Unity Technologies, San Francisco, USA) and was presented to participants on a 27-inch computer monitor (1920 × 1080 px).

The game area corresponded to a 120 m × 90 m field, fenced off on each side with walls that prevented the HA and the TAs from leaving the game field. TAs were represented as spherical cows, each with a horned cubical head and a black and white textured body (see figure 1*a*). The walls exerted a repulsive force of 4 N on the TAs (of unit mass) upon collision, thus preventing the TAs from constantly moving along the walls of the game field. The HA could influence the TA by coming within a radius $d_{i}=10$ m of the TA, with the TA’s movement dynamics defined as


(6.1) $$\ddot{x}(t)=\alpha_{r}\frac{x(t)-y(t)}{\|x(t)-y(t){\|}^{2}}-\beta \dot{x}(t).$$

Here, $𝐱(t)\in {\mathbb{R}}^{2}$ and $𝐲(t)\in {\mathbb{R}}^{2}$ are the positions of the TA and HA with respect to the centre of the containment zone, respectively, and the dots above the variables refer to the standard time derivatives. β is the drag coefficient set to $0.2\;s^{-1}$ and $\alpha_{r}$ is a constant set to 20 $m^{2}\;s^{-2}$ when the HA is within the influence area of the TA and 0 otherwise (values set heuristically to maintain similarity across congruent environments; see [34,35]). This results in the TA moving in the direction directly away from the HA approach vector when the HA enters the influence area. Note that the maximum repulsion the TA could experience was clamped at 30 N.

As noted above, the containment zone was specified by a red translucent, non-rigid-body circular area with a radius of 4 m and was always located at the centre of the game field. Both the TA and the HA could freely pass through (in and out) the containment zone without being impeded by the containment zone.

Participants controlled the direction of motion and angular orientation of the HA using keyboard and mouse controls, respectively, similar to a classical first-person shooter game (e.g. Wolfenstein 3D). Using the W, A, S and D keys, the HA could move forward, left, backwards and right, respectively. Upon pressing two keys—WD, WA, SD and SA—simultaneously, the HA would move diagonally with respect to the forward direction. The HA’s speed was fixed at 5 m s^−1^. The HA’s orientation could be rotated to the right or to the left by moving the mouse in a corresponding transverse (left–right) direction. The HA’s field of view spanned 60° in the vertical direction and 97.6° in the horizontal direction. Note that the HA’s head movement (camera orientation) was decoupled from its body movement (translation) allowing participants to visually explore the environment without moving around.

Participants were required to complete N trials, with N=20 for the single-target herding experiment and N=24 for the multi-target herding experiment. The first N/4 trials were included as practice trials (i.e. five practice trials in the single-target herding experiment and six practice trials in the multi-target herding experiment). N different initial conditions were pre-specified and used for all participants, with initial conditions for the first quarter (practice trials) and the last three-quarters (experimental trials) randomly ordered across this respective division, for each participant. Each initial condition included different (x,y) game field positions of the HAs and TAs (never located within the containment zone), as well as a different initial HA heading angle. For each trial the HA and TA game field positions, heading angles (defined with regard to the centre of the game field) and movement velocities were recorded at 50 Hz.

A game trial started with the HA and TA positioned according to the pre-determined initial conditions and came to an end when the TA was corralled within the containment zone and its speed was less than 0.1 m s^−1^. This required the HA to stop influencing the TA prior to the TA reaching the containment zone, such that the TA slowed down and came to rest within the containment zone. That is, if the HA continued to influence the TA up until it entered the containment zone or after it entered the containment zone, the TA would simply pass through the containment zone without the trial ending.

### 6.2. General procedure

After arriving at the laboratory, participants were seated at a desk with the computer monitor, keyboard and mouse used for the study positioned directly in front of them. Participants then read and signed a consent form and completed a demographic survey. Participants were then informed that the study was investigating and modelling human navigation and herding behaviour and that they would be completing a simple herding task that required them to control a virtual HA to locate and corral one or more TAs into the red containment zone. Participants were not instructed about how to best corral and herd the TA but were instructed to complete the trials in the shortest time possible. After participants indicated they understood the task, they completed all trials in a single session.

### 6.3. Single-target herding experiment

#### 6.3.1. Participants

Twenty-four participants from Macquarie University, Sydney, Australia, were recruited for the study. The participants were between 18 and 36 years of age (*M* = 20.75, s.d. = 3.64). Twenty participants self-identified as female and four as male. Twenty-two participants were right-handed, one left-handed and one identified as ambidextrous. The participants completed all 20 trials in a single session lasting 20–25 min.

#### 6.3.2. Data pre-processing

As noted above, the first five trials were considered practice trials and were excluded from further analyses. Six highly erroneous human trajectories were also manually discarded from analysis, in which the human participant either (i) continuously overshot the TA or containment zone and had to circle back around (and around) the field to re-herd the TA, or (ii) was the only participant to circle around the field in a direction opposite to the rest of participants. These six trials represented only 1.67% of the total number of experimental trials recorded.

Recall that the aim of the current study was to model the navigational trajectories of the participants as they moved towards, approached and then incorporated the TA into the containment area. It is important to note that at the start of a trial if the TA(s) were not within their HA’s initial field of view, participants typically rotated their HA’s head or body around to perform an initial visual scan for the location of the TA(s). However, given the above focus of the current work and the trivial nature of this scanning behaviour, an analysis or model of this behaviour was not considered here.

It is also important to note that at the end of the trial, some participants retreated away from the containment zone after successfully incorporating the TA(s) into the containment zone. To exclude this non-herding behaviour from model parametrization, the end of the HA’s TA-directed navigational and corral movements was defined as the last moment in time the HA influenced the last TA herded (or the TA in the single-target experiment).

#### 6.3.3. Train–test split

Out of the 15 trials, the first 12 were chosen as the training set on which to parametrize our movement model and the last three were chosen as test trials on which to evaluate the simulated HA against. Given that trials were presented to the participants in a randomized order, the training and test split were also randomized. Furthermore, out of the three test trials, it was observed that in one trial approximately half (*n* = 10) participants took a route tending towards the top half of the game field, and the other group (*n* = 14) took a route tending towards the bottom. This was because the HA’s initial position, centre of the containment zone and TA’s initial position were almost perfectly aligned (angle = 179.5°). This further corroborates the previous observation that the initial heading angle of the human HA does not play a role in the route selection, as all 24 human HAs had the same initial heading direction. This particular trial was thus split into two subtrials corresponding to the top and bottom groups, respectively; refer to the bottom panel of figure 4 for illustrations.

#### 6.3.4. Mean human trajectories and confidence bounds

Once the post-navigational transients were removed from the participant HA trajectories, time-normalized mean human HA trajectories were generated for each of the 20 initial conditions. These were obtained by first resampling the participants’ HA (x,y) trajectories to 1000 points for each trial and then calculating the mean (x,y) value at each time index for the corresponding initial condition.

To assess the variation in human/participant trajectories within the model test set, a 90% confidence interval around the average human HA trajectory was calculated. The interval, symmetrically positioned, was determined by multiplying 1.645 times the s.d. of point-to-point distances between individual participant trajectories and the mean human trajectory for each initial condition.

#### 6.3.5. Definition of terms in the navigational model

In order to accurately capture the movement of human participants, the repulsive and attractive coupling terms appearing in the model (§2.1.2) had been defined according to our conceptualizations of the requisite behaviours. Specifically, the term $\psi_{G}$ representing the attractive coupling between the HA heading direction and the angular position of the offset location ${𝐱}_{c}=𝐱+𝐜$, where $𝐜:=c\frac{𝐱}{\left\|𝐱\right\|}$, was defined as


(6.2) $$\psi_{G}(x,y,\phi ,\theta_{G}):=-k_{g}(\phi -\theta_{G})(e^{-c_{1}d_{c}(x,y)}+c_{2}),$$

where $\theta_{G}$ is the angle of the offset location with respect to the vertical axis centred on the HA, $k_{g}$ reflects the strength at which the HA’s heading direction is attracted towards the offset location, $c_{1}$ and $c_{2}$ are positive constants and $d_{c}\left(𝐱,𝐲\right)=\|𝐱+𝐜-𝐲\|\in {\mathbb{R}}^{+}$ is the distance between the offset location and the HA. Note that $\left(e^{-c_{1}d_{c}\left(𝐱,𝐲\right)}+c_{2}\right)$ ensures that the attractive coupling to the offset target location decays as a function of $d_{c}\left(𝐱,𝐲\right)$ but does not completely vanish.

**Figure 7.**
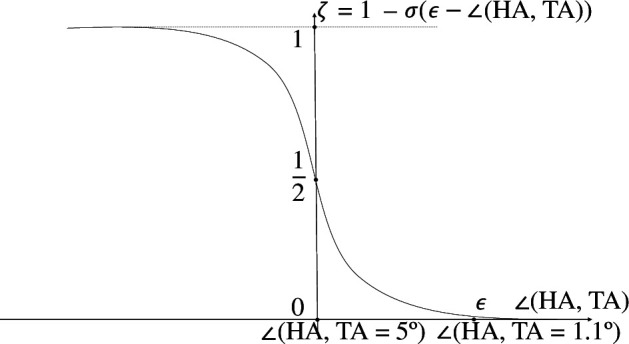
Justification of choice of $\epsilon =5^{o}$. ∠(HA,TA) is the angle between the HA and the TA, subtended at the centre of the containment zone, equal to $\cos^{-1}(\frac{𝐱\cdot 𝐲}{\left\|𝐱\|\|𝐲\right\|})$. This brings the influence of the target-as-a-repeller term towards 0 as the simulated HA approaches 1.1°. ϵ−∠(HA,TA) is plotted on the x-axis and the function ζ on the y-axis. ζ=0 when ∠(HA,TA)=5°.

The term $\psi_{T}$, which repels the HA from the TA’s current location, is defined as


(6.3) $$\psi_{T}(x,y,\phi ,\theta_{T}):=\zeta (x,y)k_{o}(\phi -\theta_{T})e^{-c_{3}|\phi -\theta_{T}|}e^{-c_{4}\|x-y\|},$$

where $\theta_{T}$ is the angle between the TA and vertical axis centred on the HA, $k_{o}$ represents the strength at which the HA’s heading direction is repelled away from the location of the TA and $c_{3}$ and $c_{4}$ are other positive constants. The exponential modulation terms ensure that the repulsion decays rapidly as the HA faces away from the TA and as the two agents get further away. The function ζ(𝐱,𝐲) in equation (6.3) is a sigmoidal function chosen as


(6.4) $$\zeta (x,y)=1-\sigma (\epsilon -\cos^{-1}(\frac{x\cdot y}{\left\|x\|\|y\right\|})),$$

with $\sigma (z)=1/(1+e^{-z})$, ensuring that the negative coupling term in equation (6.3) decays towards zero as the HA approaches the TA. The parameter ϵ was set to $5^{o}$ (as indicated from the human trajectory data, at $1.1^{o}$ the HA first influences the TA) measured from the line joining the centre of the containment zone and the TA (more simply the angle between 𝐱 and 𝐲—see figures 2a,b,7). ζ thus defines when the transition between the approach phase and the corral phase occurs.

The term $\psi_{C}$, which repels the HA away from the containment zone C (the centre of which, ${𝐱}_{C}$, is stationary at the origin), is defined as


(6.5) $$\psi_{C}(x_{C},y,\phi ,\theta_{C}):=k_{o}(\phi -\theta_{C})e^{-c_{5}|\phi -\theta_{C}|}e^{-c_{6}\|x_{C}-y\|},$$

where $\theta_{C}$ is the angle between the centre of the containment zone and the vertical axis centred at the HA, and $c_{5}$ and $c_{6}$ are positive constants. The term $\psi_{C}$ repels the HA away from the containment zone at all times.

#### 6.3.6. Trajectory measures and comparison analysis

This model, complete with all degrees of freedom defined, was thus able to generate simulated trajectories. To compare simulations with human data, the errors for individual humans in the trials corresponding to the test dataset were calculated as the DTW [31,32] distances from the mean human trajectory, per trial. These errors were ranked and the 90th-percentile error was compared with the error of the simulation with respect to the mean human trajectory. The DTW distance was employed as it allows one to assess the difference between two time-series trajectories independently of differences in movement speeds or sampling frequency. This was crucial here as human participants could actually start and stop their movement while the simulated trajectories were made a constant speed.

#### 6.3.7. Model parametrization and simulations

To parametrize the model on mean human data, an algorithm was required to minimize the DTW distance between the simulated and mean human data, through an exploration of the parameter space. Sequential least squares quadratic programming (SLSQP) [54,55] was employed to find this minimum. This algorithm was used because it is one of the methods that consistently finds the optimum for similar problems in a reasonable number of steps [42]. In particular, the scipy.optimize.minimize(method=‘SLSQP’) Python function was used. For each such trial in the model training set, the heading angle input to the simulation was taken as the tangent of the mean human trajectory near the HA’s initial position.^1^ The initial positions of the HA and TA for the simulations corresponded to the respective inputs to the human HA trials.

Ten different uniformly randomly chosen initial values for $c_{1}$ and $c_{2}$ were chosen in the range (0.1, 0.9) each, with identically chosen exploration limits. The median of the parameters output by each of these 10 runs was taken, and then the mean, median and s.d. thereof across trials. Using these latter median values, the parametrization algorithm was run for the parameters $k_{g}$ and $k_{o}$, which were chosen with initial values in the range (35, 50) and (150, 220), respectively, with exploration bounds (25, 60) and (150, 250). This procedure was identically reproduced for $c_{5}$ and $c_{6}$ with ranges and bounds (0.1, 1) each.

Once the optimum parameters for each initial condition were chosen, the overall median values per parameter were taken and then entered into the simulated HA following the model equation (2.2) for its heading and constant speed. Per trial, the average initial position and heading of the human participants and the initial TA position were fed into the simulation set-up. The resulting simulation trajectories were pre-processed by discarding the last 20% since the simulated HA remained in place while the human HAs withdrew from the TA at the end. Independent samples and Bayesian *t*-tests were performed on the trajectory measures across human and simulation data, for the model validation set.

The offset location’s distance from the TA c was set to 8.5 m (since the repulsion distance was 10 m) and the damping constant b to 3.5 $s^{-1}$ in the simulations, as that resulted in good fits. The HA had been run at the only non-zero speed available to the human participants, 5 m s^−1^.

### 6.4. Multi-target herding experiment

#### 6.4.1. Participants and set-up

Twenty-one participants from Macquarie University were recruited for the study. The participants were between 18 and 33 years of age (*M* = 21.05, s.d. = 3.54). Sixteen participants identified as female, four as male and one as non-binary or third gender. Twenty participants were right-handed and one left-handed. The participants completed all 24 trials in a single session lasting 25–30 min. None of the data were discarded, and no pre-processing was performed. The aim of this experiment was to infer the TS strategy adopted by human actors in this task context.

Once the TS strategy was inferred from this first dataset, a new set of 10 participants recruited from the Scuola Superiore Meridionale, Italy, were tasked with playing the same video game, the only difference being that a different set of initial conditions was used, with the number of trials unchanged. This second dataset was used to validate the results of the first analysis. Ten participants were recruited, aged between 25 and 32 years (*M* = 27.80, s.d. = 2.32). Three participants identified as female and seven as male. The 10 participants were right-handed.

#### 6.4.2. Definition of policies

To infer the actual TS strategy adopted by human participants, several different TS policies were tested. Four different general types of TS strategies have been defined. In each of these types of strategies, the TAs were ranked in the appropriately defined order according to their respective criteria.

*Initial condition policies*—where the entire TS sequence is defined by the initial state of the environment prior to herder movement or target engagement

— closest or furthest linear distance from herder: ‘Closest/Furthest Distance From Herder’
— closest or furthest angular distance from herder: ‘Closest/Furthest Angle From Herder’
— closest or furthest linear distance from the containment zone: ‘Closest/Furthest From Containment Zone’.

*Successive policies*—where the first TA that is selected is defined by the initial state of the environment, but then subsequent TSs are made successively at the time of target engagement (but before the target is corralled into the containment zone)

— successive closest or furthest linear distance from herder: ‘Successive Closest/Furthest Distance from Herder’
— successive closest or furthest angular distance from herder: ‘Successive Closest/Furthest Angle from Herder’.

*Dynamic policies*—similar to successive policies, but subsequent TSs are made dynamically when the preceding TA has been corralled

— dynamic closest or furthest linear distance from herder: ‘Dynamic Closest/Furthest Distance from Herder’
— dynamic closest or furthest angular distance from herder: ‘Dynamic Closest/Furthest Angle from Herder’.

*Complex policies*—a combination of the above and/or an above policy that includes a target grouping or clustering function. This target grouping function takes into account the vicinity (in terms of angular distance) of TAs. In case of vicinity in between TAs, precedence is taken for the TA that is further in linear distance from the containment zone. In the case of absence of vicinity in between TAs, as well as in selecting the first TA and subsequent TAs/clusters, the respective base (initial, successive or dynamic) policy was followed according to the respective angular (‘Angle’) or linear distance (‘Distance’) classifier. Vicinity had been defined empirically as the angle between any two TAs less than 18.9°.

— ordering of TA selection on the basis of initial conditions: ‘Initial Collinear Angle/Distance’

— ordering of TA selection successively: ‘Successive Collinear Angle/Distance’

— ordering of TA selection dynamically: ‘Dynamic Collinear Angle/Distance’.

#### 6.4.3. Policy testing

To test the accuracy of the predictions of our defined policies, the following steps were performed. First, the actual order in which the TAs were chased was recorded, per participant. Then this ordering was compared to the ordering predicted by our defined policies, based either on the initial positions of the HA and TA or on the entire positional time series per trial. These two different types of input to our policy model function correspond to the distinction between the initial/successive and dynamic/complex policies.

If (and only if) the predicted ordering perfectly matched the observed ordering, the score assigned to the policy behind the prediction was increased by one. Going through all participants and trials as such, the final score was normalized to give a percentage value.

Such percentage scores could be calculated using one of two ways. One would consider mutual exclusivity among the policies such that the total score across all the tested policies and the exceptions sum to 1. If an observed ordering matched a policy’s prediction, the score of that policy would be incremented by one and that of the others by zero. The other way (non-mutually exclusive) would be to calculate all scores independently of other policies, so that every time an observed ordering matched a policy’s prediction, the latter’s score would be incremented, regardless of how many different policies could match the observation.

#### 6.4.4. Generalization of movement model

To be able to generate simulated HA trajectories on the game plane that followed the inferred SCA policy, the navigational herding model, equation (2.2) from the single-target herding experiment was modified as follows to account for multiple TAs:


(6.6) $$\ddot{\phi }=-b\dot{\phi }+\psi_{G}+\psi_{T}+\sum_{i=1}^{N}\psi_{O_{i}},$$

where $\psi_{G}$ and $\psi_{T}$ take the same form as in equation (2.2) but apply only to the currently targeted TA, and $\sum_{i=1}^{N}\psi_{O_{i}}$ is the sum of repulsive terms each taking the form of $\psi_{C}$ from equation (2.2). More specifically, each term $\psi_{O_{i}}$ is of an identical form as $\psi_{C}$ and $\sum_{i=1}^{N}\psi_{O_{i}}$ now encompasses not only the containment zone as a repulsive obstacle but also the non-targeted TAs as repulsive obstacles. For example, for a non-targeted TA $O_{j}$ at position ${𝐱}_{j}$ and angle $\theta_{O_{j}}$ from the vertical,


(6.7) $$\begin{array}{rl} \psi_{O_{j}} & =\psi_{O_{j}}(x_{j},y,\phi ,\theta_{O_{j}})\\ & =k_{o}(\phi -\theta_{O_{j}})e^{-c_{5}|\phi -\theta_{O_{j}}|}e^{-c_{6}\|x_{j}-y\|}. \end{array}$$

As the containment zone and non-targeted TAs are each modelled by this repulsive form,


(6.8) $$N=\underset{for\;the\;containment\;zone}{\underbrace{1}}+\underset{non\text{-}targeted\;TAs}{\underbrace{\left(N_{TAs}-1\right)}}.$$

The parameters that appear in each of the $\psi_{O_{i}}$ terms are identical to the parameters for the $\psi_{C}$ term in the single-target herding experiment. Similarly, all other parameters in equation (6.6) were the same as in equation (2.2). The SCA TS policy was evaluated once every 0.25 s to determine the currently targeted TA.

#### 6.4.5. Validity of simulated herder agent

To test the applicability of the inferred TS policy, simulations were made by coupling this policy to the single-target movement model (as explained in §3.1.3). The simulations were then evaluated against the human data by first constructing a nonlinear (specifically, square root) heat map of the spatial occurrences of trajectory points in square 5 m × 5 m bins. The resulting heat map was filtered through for bins with a value (obtained through trial-and-error) greater than 10, to eliminate bins with only single trajectories passing through, to obtain a binary map.

The percentage of any given trajectory falling through this binary map was calculated as the number of bins the trajectory passed through in the binary map for a given set of initial conditions, normalized by the total number of bins assigned to that given trajectory. This value was called the ‘binary trace’ of a trajectory. The ‘weighted trace’ of a trajectory was defined as the similarly normalized sum of the values of the nonlinear heat-map bins through which that trajectory had passed. The traces of each human participant were calculated and the mean and s.d. was taken across all trials.

## Data Availability

Data can be accessed through [56]. Accompanying code and game programs can be accessed through GitHub [57]. Supplementary material is available online [58].

## Footnotes

^1^
Tangent between the 200th and 50th point, as that is approximately when the human HA starts moving.
